# Selection and Validation of Reference Genes for qRT-PCR Gene Expression Analysis in *Kengyilia melanthera*

**DOI:** 10.3390/genes13081445

**Published:** 2022-08-14

**Authors:** Junming Zhao, Jian Yang, Xiaoyun Wang, Yanli Xiong, Yi Xiong, Zhixiao Dong, Xiong Lei, Lijun Yan, Xiao Ma

**Affiliations:** 1 College of Grassland Science and Technology, Sichuan Agricultural University, Chengdu 611130, China; 2 Sichuan Academy of Grassland Science, Chengdu 611731, China

**Keywords:** *Kengyilia melanthera*, reference genes, real-time quantitative PCR, gene expression

## Abstract

*Kengyilia* is a newly established genus. Most species in this genus survive in hash environment, which might be an indicator of an acquirement of stress resistance genes and the potential for molecular breeding in *Triticeae* species. Quantitative real-time PCR (qRT-PCR) is a widely used technique with varied sensitivity heavily dependent on the optimal level of the reference genes. *K. melanthera* is a typical psammophyte species which has high drought resistance. The reference genes of *K. melanthera* are not yet reported. This study aims to evaluate the expression stability of 14 candidate reference genes (*EF1A, GAPDH, ACT1, UBI, TUBB3, TIPRL, CACS, PPP2R1B, TUBA1A, EIF4A1, CYPA3, TCTP, ABCG11L,* and *FBXO6L*) under five treatments (drought, heat, cold, salt, and ABA) and find the most stable and suitable one even upon stressed conditions. The software NormFinder, GeNorm, BestKeeper, and RefFinder were used for data analysis. In general, the genes *CACS* and *PPP2R1B* are concluded to have the best overall performance under the various treatments. With the ABA treatment, *TCTP* and *TIPRL* show the best stability. *CACS* and *TCTP*, as well as *TIPRL* and *CYPA3*, were most stable under the treatments of cold and salt, respectively. *CACS* and *FBXO6L* were ranked the highest with the heat treatment and drought treatment, respectively. Finally, the Catalase-1 (*CAT1*) gene was used to verify the reliability of the above reference genes. Accordingly, *CAT1’s* expression pattern remained unchanged after normalization with stable reference genes. This study provides beneficial information about the stability and reliability of potential reference genes for qRT-PCR in *K. melanthera*.

## 1. Introduction

There are about 26 species and six varieties of *Kengyilia,* a genus in the tribe Triticeae established in 1990 [1]. *K. melanthera* is a self-pollinated perennial grass species with allohexaploid. It is a typical psammophyte species, mainly distributed in sandy river banks, dunes, and sandy meadows of the Qinghai–Tibet Plateau at an altitude of 3300–4750 m [2]. As such, *K. melanthera* has strong drought and cold resistance. It can be used as a grass for plateau desertification control and ecological restoration, or as plateau forage because of its high biomass and nutritional quality. In addition, some studies reported that *K. melanthera* has a strong resistance to wheat head scab [3]. *Kengyilia* and other Triticeae perennials have a vast genetic reservoir, which might be used to improve annual cereals [4]. Wheat (*Triticum aestivum*) is one of the major staple crops in the world, of which the yield is limited by abiotic stress and biotic stress [5]. Among cultivated wheat, the breeding potential is already exhausted as there is an increasingly narrow range of genetic variation [6]. At present, certain genes of related species are transferred to wheat to improve its yield and stress resistance [5,7,8]. We consider it necessary to explore *K. melanthera’s* resistance genes and related regulatory pathways, given their potential for wheat improvement.

Gene expression analysis is a useful tool in exploring the mechanisms of stress resistance in *K. melanthera*. Quantitative real-time polymerase chain reaction (qRT-PCR) is a frequently used technique to examine the gene expression level with the strength in fast reaction, precision, specificity, sensitivity, and repeatability [9,10,11,12]. Absolute and relative quantification are two methods of quantitative gene expression [13,14]. However, it is usually unnecessary to express quantitative data as an absolute copy number [14]. Therefore, relative quantification is more commonly applied [13]. In most cases, the reliability of qRT-PCR is affected by multiple factors, including RNA quantity and quality, the efficiency of cDNA synthesis, the quantity of the starting DNA template, and so on [10,12,15]. Hence, to compensate for the above difference, it is inevitable to have a requirement for reliable reference genes for normalization.

The ideal reference genes are supposed to exhibit the least expression level difference under different environmental stresses or in different tissues, organs, and developmental stages [9,11,15]. In qRT-PCR analysis for plant species, common reference genes include *Actin*, Polyubiquitin (*UBQ*), Elongation factor-1a (*EF-1α*), α-Tubulin (*TUA*), *18S rRNA*, β-Tubulin (*TUB*), Cyclophilin (*CYP*), and Glyceraldehyde-3-phosphate dehydrogenase (*GAPDH*) [16,17,18]. However, there are quite a few studies reporting that the expression level of these reference genes is not stable under different conditions [19,20,21,22]. Inappropriate selection of the unsuitable reference genes can lead to significant mistakes in results [23]. Therefore, identifying ideal reference genes for a particular species and understanding their performance under different conditions are critical for delivering reliable gene expressional analytical results [13,24].

Currently, no reference genes have been described for *K. melanthera*. Herein, the expression stability of 14 reference gene candidates in leaves under different experimental situations (drought, heat, cold, salt, and abscisic acid) was identified and verified by comparison to the Catalase-1 (*CAT1*) gene. This work aims to contribute to the future exploration and utilization of *K. melanthera’s* resistance genes.

## 2. Materials and Methods

### 2.1. Plant Materials and Treatments

*K. melanthera* seeds were kindly provided by Sichuan Academy of Grassland Sciences (Chengdu, China). Seeds were planted in plastic pots (20 × 15 × 5 cm) with quartz sand. A total of 1.5 g of seeds were used for each pot. Hoagland nutrient solution was applied. The pots were placed in a growth chamber at 25 °C/20 °C (day/night). A 12 h photoperiod was set for the growth chamber. Twenty-one-day old plants were used for all experiments. Five treatments were set up for the screening of the reference genes. Each treatment has three biological replicates. In abscisic acid (ABA) treatment, the Hoagland nutrient solution was added with 50 µM ABA. In cold and heat treatment, the plants were placed in an incubator with a temperature setting of 4 °C or 38/33 °C (day/night), respectively. In salinity treatment, the Hoagland nutrient solution supplemented with 250 mM NaCl was applied. In drought treatment, PEG6000 at 20% concentration was performed. Finally, the leaf samples were collected at five time points, respectively—0, 12, 24, 48, and 96 h post treatment. The harvested samples were stored in a minus 80 °C lab freezer.

### 2.2. RNA Extraction and cDNA Synthesis

The RNA extraction kit (Monad Biotech, Suzhou, China) was used for the total RNA extraction following the manufacturer’s instructions. BioPhotometer (Eppendorf, Hamburg, Germany) measurement and 1% agarose gel were conducted to examine the RNA concentration and integrity. An absorbance ratio of A260/A280 ranging from 1.8 to 2.2 and A260/A230 ratio equal to 2.0 were preferred. Based on the recommendation of the *Evo M-MLV* RT Mix Kit (Accurate Biotech, Changsha, China), 0.8 µg total RNA was measured to perform the cDNA synthesis.

### 2.3. Primer Design

Fourteen reference gene candidates and one target gene were selected and named according to the sequence similarities to known genes (Table 1). These genes were obtained from our full-length transcriptome data (accession number: PRJNA735213) of *K. melanthera* by BLAST search using reported gene sequences. The Primer-BLAST tool by NCBI (https://www.ncbi.nlm.nih.gov/tools/primer-blast/, (accessed on 1 May 2021)) was used for primer design. The principles of primer design were as follows: annealing temperature at 58–62 °C (optimal Tm was 60 °C), primer length at 18–25 bp, GC content at 40–60%, and the length of amplification product between 80–200 bp. Primer specificity was detected by conventional PCR and 2% agarose gel electrophoresis.

### 2.4. Quantitative RT-PCR Amplification

CFX96 PCR detection system (Bio-RAD, Hercules, CA, USA) was used to perform qRT-PCR. The 10 µL final reaction volume included 5 µL 2 × SYBR Green Premix (Monad Biotech, Suzhou, China), 1 µL cDNA, 1 µL of forward and reverse primer (final concentration of 0.2 µmol·L^−1^), and 3 µL ddH_2_O. The PCR program was set as following: pre-denaturation at 94 °C for 20 s, followed by 40 cycles of denaturation at 94 °C for 10 s, and annealing at 60 °C for 20 s. To verify primer specificity, T_m_ and melting curves were analyzed between 65 °C–95 °C, and fluorescence values were detected at each 0.5 °C increase. All samples used for qRT-PCR analysis were set with three technical replicates. Standard curves for each candidate reference gene were constructed to determine PCR amplification efficiency and regression coefficients (R^2^), and the data were further analyzed by CFX Manager Software 3.1.

### 2.5. Analysis of Reference Gene Candidates’ Expression

The stability of 14 candidate reference genes was evaluated by GeNorm [25], NormFinder [26], BestKeeper [27], and online tool RefFinder [28]. Prior to GeNorm and NormFinder analysis, the raw Cq values need to be converted into relative quantities according to the formula 2^−ΔCq^ (ΔCq = each corresponding Cq value − lowest Cq value) [29]. GeNorm determined the stability of reference genes by calculating M values; the reference genes with better stability have smaller M values. GeNorm can also calculate pairwise variation (V). When the V_n_/V_n+1_ value ≤ 0.15, the number of suitable reference genes is “n” [25]. NormFinder selected the most suitable reference gene by calculating the stability value of candidate gene expression. A low stability value indicates that the gene is stable [26]. BestKeeper calculated standard deviation (SD) and coefficient of variation (CV) by raw Cq values. The more stable reference gene had the lower SD value [27]. RefFinder can generate a comprehensive ranking based on the analysis results of GeNorm, BestKeeper, NormFinder, and Delta Ct.

### 2.6. Validation of Reference Genes

The two most stable reference genes, alone and in combination, and the least stable reference gene were used to normalize the expression of the target gene, namely *CAT1*. The results were calculated by 2^−ΔΔCq^ method [30]. SPSS 22 (IBM, Armonk, NY, USA) was used for statistical significance analysis.

## 3. Results

### 3.1. Primer Specificity and Amplification Efficiency

In this study, the sequences of 14 reference gene candidates and one target gene were extracted from the full-length transcriptome of *K. melanthera*. All had >90% identity with the sequences of homologous genes registered in the NCBI, and their E values were zero (Table S1). The results of PCR amplification and 1% agarose gel electrophoresis (matching the expected amplicon size and single amplicon fragments on the gel) further proved the sequence accuracy (Figure S1). In addition, the melting curve of each pair of primers had only one peak, indicating their specificity (Figure S2). The primer amplification efficiency was in the range of 91.1% and 106.5%, and the regression coefficients (R^2^) was in the range of 0.985 and 0.999 (Table 1). Therefore, our qRT-PCR data was reliable.

### 3.2. Expression Profile of the 14 Reference Gene Candidates in Response to Different Treatments

**Figure 1:**
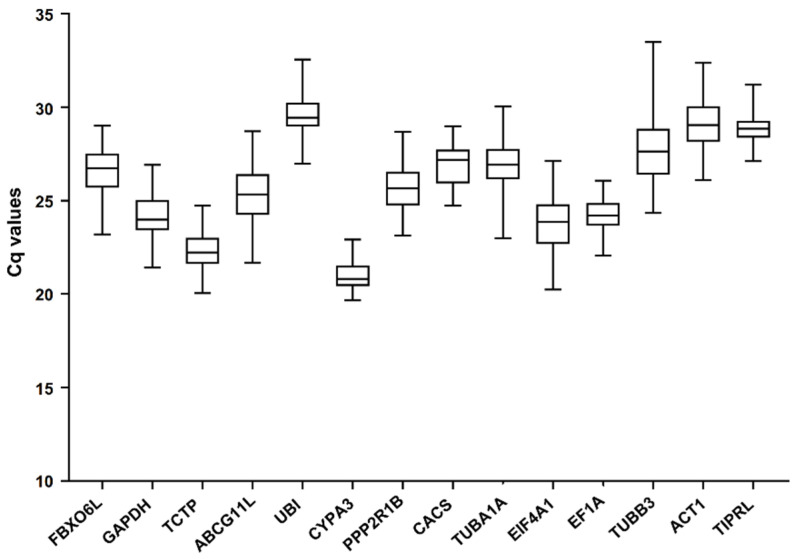
The Cq values for 14 candidate reference genes in all samples.

The expression abundance of 14 reference gene candidates in all samples are demonstrated in Figure 1. They varied among different samples. Cq values ranged from 19.66 to 33.48. *CYPA3* had the highest expression with an average Cq value of 20.99, while *UBI* had the lowest expression with an average Cq value of 29.57. In addition, *TUBB3* had the largest range of Cq values (24.34–33.48), and that of *CYPA3* was the lowest (19.66–22.91). These results suggested that these candidate genes were not stably expressed in the stressed conditions. It is imperative to conduct a screening to find out appropriate reference genes in *K. melanthera* under certain conditions.

### 3.3. Analysis of Reference Genes Stability

A total of 14 reference candidate genes are subjected to the evaluation of the expression stability in response to five treatments with the help of NormFinder, BestKeeper, and GeNorm. The final overall ranking was calculated by RefFinder.

#### 3.3.1. GeNorm Analysis

**Figure 2:**
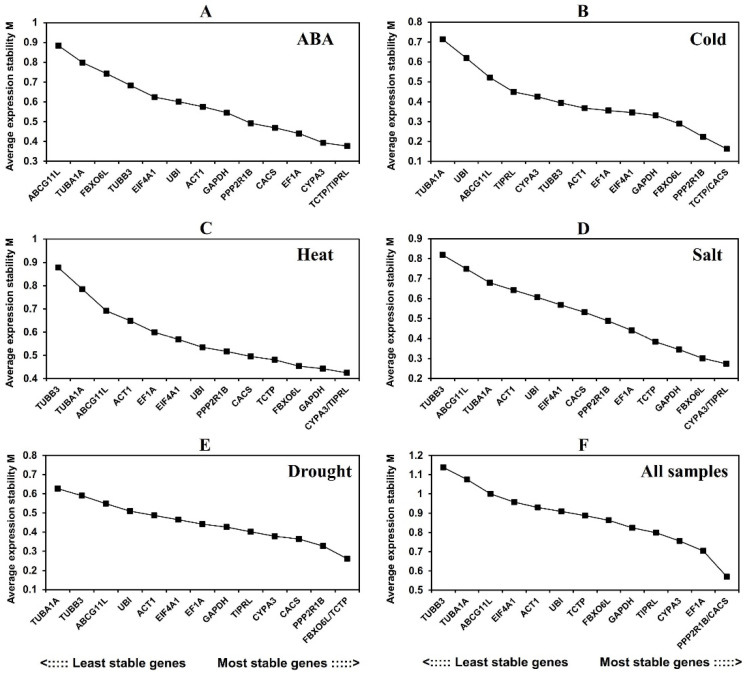
Average expression stability values (M) of fourteen candidate reference genes calculated by GeNorm.

GeNorm ranked each reference gene candidate by calculating the stable value M. The genes with M value < 1.5 were regarded as stable expression, and the ones with the lowest M value were counted as the most stable ones. The M values of 14 reference gene candidates in response to different conditions were all <1.5 (Figure 2). Under ABA treatment, *TCTP* and *TIPRL* had the lowest M value (M = 0.377) and the most stable expression. Under cold treatment, *TCTP* and *CACS* expression was the most stable (M = 0.163). Under heat treatment and salt treatment, *CYPA3* and *TIPRL* showed the most stable expression (M = 0.425; M = 0.274). Under drought treatment, *FBXO6L* and *TCTP* had the lowest M value (M = 0.261). In all samples, *PPP2R1B* and *CACS* showed the best stability (M = 0.570), followed by *EF1A* and *CYPA3*.

**Figure 3:**
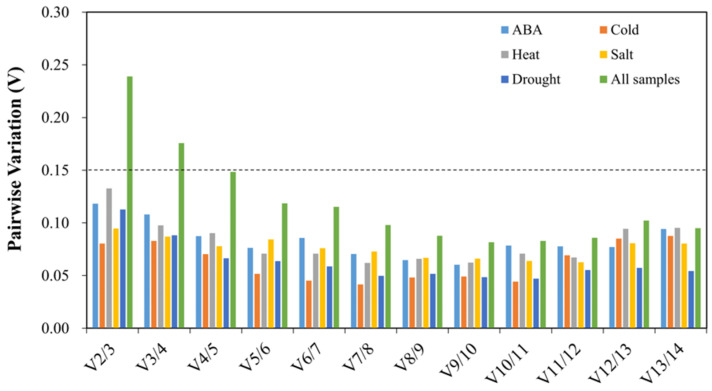
Pairwise variation (V) of fourteen candidate reference genes. Dashed lines represent the threshold (0.15).

GeNorm also calculated the paired variation (V_n/n+1_) value to determine the optimal number of reference genes for the normalization of target gene expression. If the V_n/n+1_ value is <0.15, the optimal number of reference genes is n; if not, another reference gene should be brought into consideration [25]. In this study, V_2/3_ values were <0.15 under each treatment alone, while V_2/3_ and V_3/4_ values were >0.15 and V_4/5_ values were <0.15 in all samples (Figure 3). The results showed that it was adequate to use the combination of two reference genes in a separate treatment, but the combination of four reference genes was needed in the synthesis of all treatment samples.

#### 3.3.2. NormFinder Analysis

**Figure 4:**
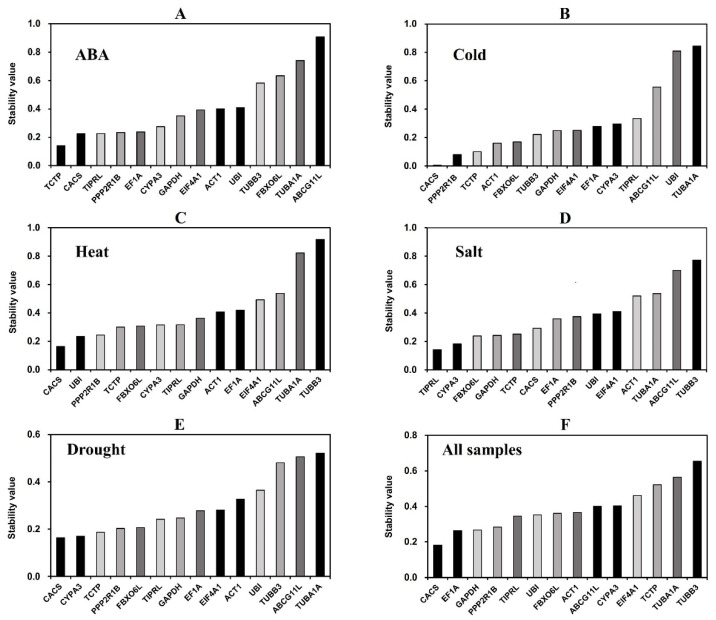
Expression stability of the fourteen candidate reference genes calculated using NormFinder.

The expression stability values of 14 reference gene candidates were calculated and ranked by NormFinder (Figure 4). The lower the stability value indicates the higher stability of the reference gene expression. In cold, heat, drought, and all samples, *CACS* was ranked as the most stable reference gene, with stability values of 0.005, 0.165, 0.164, and 0.182, respectively. Under ABA treatment, *TCTP* was the most stable gene, with a stability value of 0.141. *TIPRL* was the most stable gene under salt treatment, with a stability value of 0.142. In various treatments, *TUBA1A* and *ABCG11L* were ranked lowest, indicating their poor stability, consistent with the results of GeNorm analysis.

#### 3.3.3. BestKeeper Analysis

BestKeeper calculated the CV and SD according to the original Cq value [27]. The lower the CV ± SD, the more stable the gene expression. The most stable genes under ABA, cold, and drought treatment were *EF1A, TIPRL* and *PPP2R1B*, respectively. The *FBXO6L* gene was identified to have the highest stability in heat and salt treatment, while its stability in ABA, cold stress, and all samples ranked in the middle. Obviously, the stability of a gene may change under different stress treatments. Among all samples, *TIPRL* was the highest stable gene. The stability of the *ABCG11L* gene ranked lowest of all treatments (Table 2).

#### 3.3.4. RefFinder Analysis

The online tool RefFinder was used to comprehensively rank 14 candidate reference genes. Based on the rankings of GeNorm, NormFinder, BestKeeper, and Delta Ct, it assigns an appropriate weight to each individual gene and calculates the geometric mean of its weight to obtain a comprehensive ranking (Table 3). Under ABA treatment, *TCTP* and *TIPRL* had the best stability. *CACS* and *TCTP* were the two best stable genes under cold treatment. *TIPRL* and *CYPA3* showed the best stability under salt treatment. In both heat and drought treatment, *CACS* and *FBXO6L* stability ranked highest. Finally, in all samples, *CACS* and *PPP2R1B* had the highest stability and *TUBB3* had the lowest. According to RefFinder’s comprehensive ranking, there were differences in gene stability under different treatments.

### 3.4. Validation of The Reference Genes Identified from This Study

**Figure 5:**
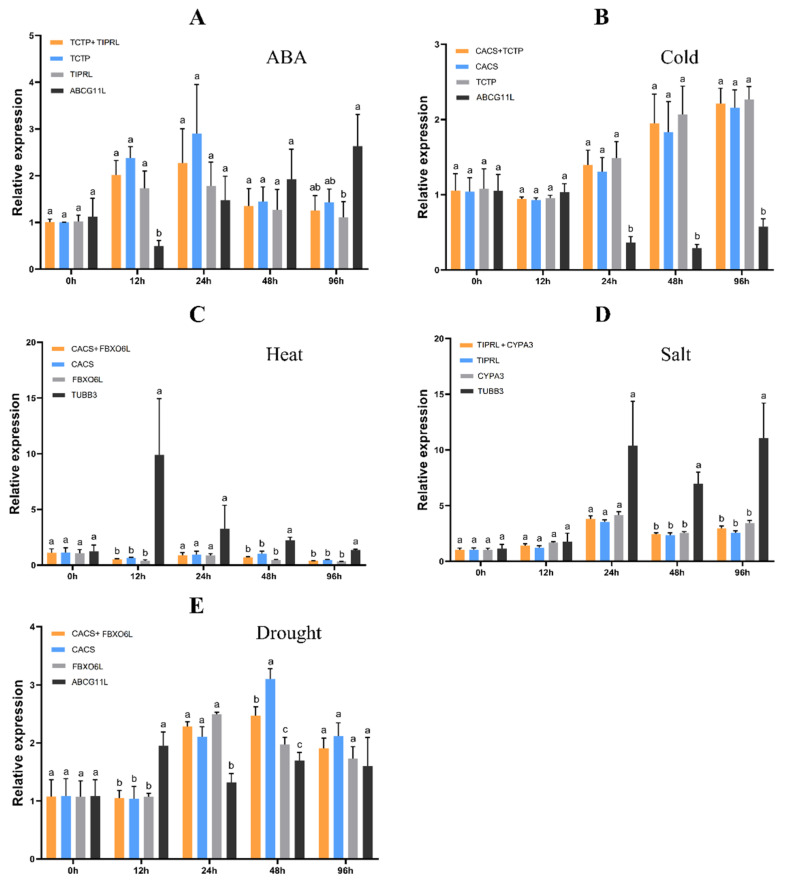
The relative expression levels of *CAT1* genes under different treatments were normalized by the two most stable genes and the least stable genes. Different letters at the same time point indicate significant differences between reference genes or combinations at the 0.05 level. The bars indicate the standard error (±SE).

CAT helps protect cells from H_2_O_2_ by breaking it down into dioxygen and water. The expression levels of the CAT gene in plants is influenced by multiple [31]. To identify the reliability of the stability ranking of reference gene candidates, the relative expression levels of the CAT1 gene were normalized using the two highest and least stable genes in the RefFinder ranking. Except for 48 h of drought treatment, there is no significant difference in *CAT1* expression using the two most stable genes as reference genes at all other time points (*p* < 0.05) (Figure 5). When the least stable gene was used as reference, *CAT1* expression was significantly different in most cases (*p* > 0.05). For each treatment, the expression pattern of *CAT1*, analyzed by the two stable reference genes, remained unchanged. Under cold treatment, *CAT1* expression showed an increasing trend (Figure 5B); under heat, it showed a decrease–increase–decrease change (Figure 5C); under ABA and salt and drought treatment, *CAT1* first showed an increase and then a decrease (Figure 5A,D,E). However, when normalized by the most unstable reference genes, *CAT1* expression patterns tended to be abnormal. These results showed that using unstable genes used the expression results of target genes as reference biases.

## 4. Discussion

*Kengyilia* is a newly established plant genus [1]. Most species in this genus grow in harsh environments [2]; therefore, having excellent stress resistance genes of great potential value for Triticeae plant breeding. qRT-PCR plays a key role in gene expression research. Appropriate reference genes greatly enhance the precision and consistency of qRT-PCR in plants [32]. In the past, most reports focused on *Kengyilia’s* classification and phylogeny [1,33,34,35], but there was no report on the screening of reference genes. Currently, only a few plant genomes are published, excluding *K. melanthera*. Furthermore, compared to the second-generation transcriptome sequencing, the third-generation full-length transcriptome sequencing holds more advantages, including longer read length and less sequencing and assembly error, which is more beneficial for the study of plants without a reference genome [36,37]. In this study, 14 reference gene candidates were used and investigated in order to find the most stable and appropriate ones in response to different treatments.

Here, three types of software (GeNorm, NormFinder, and BestKeeper) were used to evaluate the stability of 14 reference gene candidates under five different treatments. The stability of some genes was similar in the three platforms: for instance, *TCTP* had the highest while *TUBA1A* and *ABCG11L* had the lowest stability under ABA treatment; *ABCG11L*, *UBI*, and *TUBA1A* had the worst stability under cold treatment; and *TUBA1A*, *ABCG11L*, and *TUBB3* had the worst stability under heat treatment. Nevertheless, the stability of some genes differed. Under drought treatment, the highest stable genes were *FBXO6L*/*TCTP*, *CACS*, and *PPP2R1B*, respectively. This may be owning to the algorithm difference among these three software [10].

According to RefFinder’s results, the highest stable reference genes under various experimental situations may differ. *TCTP* was the highest stable gene under ABA treatment but ranked fifth under salt treatment. *TIPRL* was the highest stable gene under salt treatment; however, it ranked seventh in drought treatment and tenth in cold treatment. *CACS* was the highest stable gene under cold, heat, and drought treatment, but it ranked sixth in salt treatment. This is in agreement with other studies: for instance, *Lycoris aurea’s* highest stable genes under drought and cold treatment were *PTBP1* and *CYP2*, respectively, while *EXP1* was the most stable gene under salt, heat, and ABA treatment [38]. Hence, it is crucial to conduct screening for the appropriate reference genes under different conditions for each species [39,40].

It Is worth noting that, although *CACS* is not a commonly used reference gene, it was the most stable under cold, heat, and drought treatment, of all samples in the RefFinder ranking of this study. *CACS* are subunits of the clathrin adaptor protein (AP) complex, through which the AP complex can collect cargo and recruit other proteins involved in the formation of clathrin-coated vesicles [41]. The *CACS* gene was reported as the highest stable reference gene in watermelons (*Citrullus lanatus*) under abiotic stress (cold, salt, drought) [42], and in cucumber (*Cucumis sativus*) under both short-term and long-term heavy metal stress (Cd, Pb, Cu, Zn, Mn, Ni) [43]. *TUB* is a traditional reference gene, but its stability ranked last in this study [18]. Abnormalities also appeared when *TUBB3* was used as a reference gene to test the expression pattern of *CAT1* gene. These data suggests that conventionally and commonly used reference genes may not be a good choice for all species and all experimental conditions. New reference gene candidates can be further explored.

Some studies have shown that normalization of target gene expression using one reference gene may lead to large experimental errors [25], which can be reduced by using ≥2 reference genes. According to GeNorm analysis, V_2/3_ values were <0.15 in all treatments; therefore, only two reference genes were enough under a single treatment condition. When considering all samples, the V_2/3_ and V_3/4_ values were >0.15, and the V_4/5_ value was <0.15; hence, four reference genes were recommended. However, in the final validation, we found that stable reference genes, either alone or in combination, could also correctly reveal the expression pattern of the *CAT1* gene. Therefore, although ≥2 reference genes can increase the precision of the expression analysis, the number of reference genes depends on experimental conditions [15,40].

## 5. Conclusions

This study is the first report that reveals a series of appropriate reference genes for *K. melanthera* under different experimental conditions. The highest stable reference genes were *CACS* and *PPP2R1B* in all samples; *TCTP* and *TIPRL* under ABA treatment; *CACS* and *TCTP* under cold treatment; *CACS* and *FBXO6L* under both heat and drought treatment; and *TIPRL* and *CYPA3* under salt treatment, respectively. This study greatly facilitates the gene expression analysis of *K. melanthera* under different experimental conditions. It also paves the way for the screening for reference gene candidates in other related species.

## Figures and Tables

**Table 1:** Information of primers and amplicons for fourteen candidate reference genes and one target gene.

Gene Symbol	Primer Sequence F/R (5′–3′)	Amplicon Length (bp)	Tm (°C)	Efficiency (%)	R^2^
*EF1A*	F: TGATATGCGCCCTGTTGATGT	128	59.2	93.9	0.995
	R: GCAGCCTACAGATAACATTCCA				
*GAPDH*	F: CTGTTCTCAAACCCCTCCGT	85	60.2	93.1	0.985
	R: GATCCGGCCGAAACCATTGA				
*ACT1*	F: CCCAAGGCCAATCGTGAGAA	97	60.3	106.5	0.999
	R: CATACAGCGAGAGGACAGCC				
*UBI*	F: AACTTCAAAGGCGCAGATTCG	165	59.5	93.9	0.994
	R: TGATAGTCTTGCCTGTGAGGG				
*TUBB3*	F: GGGCATGGATGAGATGGAGTT	143	61.3	104.1	0.995
	R: GTGGCTTATGCAGCACCTCCT				
*TIPRL*	F: TGAACGAAGACACCATGCAAAC	81	59.8	99.5	0.996
	R: CAAGGTCGATCCGGTCATCA				
*CACS*	F: AAATGGCGTGGGCTCCTTATT	125	60.2	100.3	0.998
	R: TCTGATCTGCCCTCTGCTAGT				
*PPP2R1B*	F: GCTCTGATCCCGTCAGTTGT	131	59.9	99.5	0.998
	R: TGATGGAGTTCAGGCGCAAT				
*TUBA1A*	F: TCCTTGTGCCGCCTATCTTG	89	59.9	99.7	0.998
	R: AACCCAACACCCAGACACAA				
*EIF4A1*	F: GTGACCCGTGAAGATGAGAGG	189	59.7	99.7	0.992
	R: CCCTCCCCACAGACAAGAAA				
*CYPA3*	F: AAGTTGGCGTGAGTCGTGTT	91	60.2	99.1	0.999
	R: CAGTCCACCTGAAACCCTCC				
*TCTP*	F: TGCTCTGCTCTATGGTGTTCA	152	59.4	101.1	0.991
	R: CGAGGCACTGACCAAAACAC				
*ABCG11L*	F: CTACCGCCTGCTGTTCTTCA	197	60.2	92.4	0.999
	R: GCTACCCAGCAACCCAGTTTA				
*FBXO6L*	F: ACGCAGAGACAGAAACCGAG	151	60.1	91.1	0.995
	R: GCAAACAGTGCGGAAACGAA				
*CAT1*	F: GATGAGTCCTCGATGGCGTG	84	60.2	99.2	0.999
	R: CTTTGCCGATAAGAGGGGAGAA				

**Table 2:** Expression stability and ranking of 14 candidate reference genes calculated using BestKeeper. The numbers in brackets represent coefficient of variation (CV) ± standard deviation (SD).

Rank	ABA	Cold	Heat	Salt	Drought	All Samples
1	*EF1A* (1.22 ± 0.31)	*TIPRL* (0.80 ± 0.23)	*FBXO6L* (0.95 ± 0.25)	*FBXO6L* (1.34 ± 0.36)	*PPP2R1B* (1.29 ± 0.34)	*TIPRL* (2.12 ± 0.61)
2	*TIPRL* (1.51 ± 0.44)	*CACS* (1.07 ± 0.27)	*TIPRL* (1.45 ± 0.42)	*GAPDH* (1.68 ± 0.40)	*FBXO6L* (1.42 ± 0.40)	*EF1A* (2.81 ± 0.68)
3	*CACS* (1.76 ± 0.49)	*CYPA3* (1.17 ± 0.24)	*GAPDH* (2.01 ± 0.48)	*TIPRL* (1.69 ± 0.47)	*TUBA1A* (1.56 ± 0.43)	*UBI* (2.89 ± 0.86)
4	*TCTP* (1.94 ± 0.43)	*TCTP* (1.22 ± 0.27)	*TCTP* (2.07 ± 0.44)	*UBI* (1.89 ± 0.56)	*CACS* (1.63 ± 0.45)	*CYPA3* (2.9 ± 0.61)
5	*CYPA3* (2.01 ± 0.44)	*ACT1* (1.30 ± 0.36)	*EF1A* (2.22 ± 0.53)	*PPP2R1B* (1.90 ± 0.49)	*GAPDH* (1.65 ± 0.42)	*CACS* (3.32 ± 0.9)
6	*FBXO6L* (2.03 ± 0.55)	*TUBB3* (1.38 ± 0.35)	*CACS* (2.29 ± 0.61)	*CYPA3* (2.07 ± 0.43)	*ACT1* (1.78 ± 0.54)	*TCTP* (3.41 ± 0.76)
7	*ACT1* (2.10 ± 0.64)	*FBXO6L* (1.50 ± 0.38)	*UBI* (2.32 ± 0.67)	*ACT1* (2.09 ± 0.59)	*EF1A* (1.82 ± 0.44)	*FBXO6L* (3.56 ± 0.95)
8	*UBI* (2.32 ± 0.69)	*PPP2R1B* (1.67 ± 0.40)	*CYPA3* (2.43 ± 0.51)	*TCTP* (2.13 ± 0.48)	*TIPRL* (1.82 ± 0.54)	*ACT1* (3.61 ± 1.05)
9	*PPP2R1B* (2.40 ± 0.65)	*EF1A* (2.04 ± 0.48)	*ACT1* (2.65 ± 0.77)	*CACS* (2.15 ± 0.59)	*TCTP* (2.00 ± 0.47)	*GAPDH* (3.63 ± 0.87)
10	*TUBB3* (2.55 ± 0.70)	*GAPDH* (2.09 ± 0.48)	*PPP2R1B* (2.78 ± 0.70)	*TUBA1A* (2.35 ± 0.65)	*TUBB3* (2.05 ± 0.58)	*PPP2R1B* (4.06 ± 1.04)
11	*TUBA1A* (2.65 ± 0.71)	*EIF4A1* (2.20 ± 0.48)	*EIF4A1* (3.11 ± 0.75)	*EF1A* (2.66 ± 0.64)	*CYPA3* (2.12 ± 0.44)	*TUBA1A* (4.23 ± 1.13)
12	*EIF4A1* (2.72 ± 0.69)	*UBI* (2.89 ± 0.83)	*TUBA1A* (3.27 ± 0.89)	*EIF4A1* (3.18 ± 0.75)	*UBI* (2.38 ± 0.73)	*TUBB3* (4.84 ± 1.34)
13	*GAPDH* (3.03 ± 0.74)	*ABCG11L* (3.24 ± 0.75)	*ABCG11L* (4.18 ± 1.03)	*ABCG11L* (3.47 ± 0.89)	*EIF4A1* (2.77 ± 0.67)	*EIF4A1* (5.02 ± 1.2)
14	*ABCG11L* (4.35 ± 1.15)	*TUBA1A* (4.89 ± 1.20)	*TUBB3* (4.77 ± 1.37)	*TUBB3* (4.20 ± 1.19)	*ABCG11L* (3.23 ± 0.85)	*ABCG11L* (5.35 ± 1.35)

**Table 3:** Comprehensive rankings of fourteen candidate reference genes calculated using RefFinder.

Rank	ABA	Cold	Heat	Salt	Drought	All Samples
1	*TCTP*	*CACS*	*CACS*	*TIPRL*	*CACS*	*CACS*
2	*TIPRL*	*TCTP*	*FBXO6L*	*CYPA3*	*FBXO6L*	*PPP2R1B*
3	*CACS*	*PPP2R1B*	*TIPRL*	*FBXO6L*	*PPP2R1B*	*CYPA3*
4	*EF1A*	*FBXO6L*	*TCTP*	*GAPDH*	*TCTP*	*EF1A*
5	*CYPA3*	*CYPA3*	*CYPA3*	*TCTP*	*CYPA3*	*GAPDH*
6	*PPP2R1B*	*ACT1*	*PPP2R1B*	*CACS*	*EF1A*	*TIPRL*
7	*GAPDH*	*GAPDH*	*UBI*	*PPP2R1B*	*TIPRL*	*FBXO6L*
8	*EIF4A1*	*TUBB3*	*GAPDH*	*EF1A*	*GAPDH*	*TCTP*
9	*ACT1*	*EIF4A1*	*EF1A*	*UBI*	*EIF4A1*	*UBI*
10	*UBI*	*TIPRL*	*ACT1*	*ACT1*	*ACT1*	*ACT1*
11	*FBXO6L*	*EF1A*	*EIF4A1*	*EIF4A1*	*TUBA1A*	*EIF4A1*
12	*TUBB3*	*ABCG11L*	*ABCG11L*	*TUBA1A*	*UBI*	*TUBA1A*
13	*TUBA1A*	*UBI*	*TUBA1A*	*ABCG11L*	*TUBB3*	*ABCG11L*
14	*ABCG11L*	*TUBA1A*	*TUBB3*	*TUBB3*	*ABCG11L*	*TUBB3*

## Data Availability

Not applicable.

## Supplementary Materials

The following supporting information can be downloaded at: https://www.mdpi.com/article/10.3390/genes13081445/s1, Figure S1: Amplification products of the fourteen candidate reference genes and target gene.; Figure S2: Melting curves of the fourteen candidate reference genes and target gene; Table S1: Description of 14 candidate reference genes and one target gene.
