# Supplementary material for: Selection and Validation of Reference Genes for qRT-PCR Gene Expression Analysis in Kengyilia melanthera
Source: Genes (Basel). 2022 Aug 14;13(8):1445. doi: 10.3390/genes13081445 (PMC9408421; doi:10.3390/genes13081445)
Supplement: Supplementary file 1 [file genes-13-01445-s001.zip › genes-1868539-supplementary.pdf]

Supplementary Materials

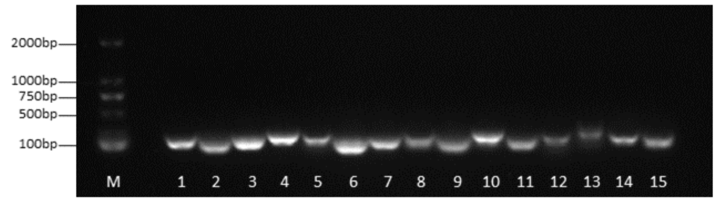

Figure S1. Amplification products of the fourteen candidate reference genes and target gene. M, DNA marker; 1, *EF-1α*; 2, *GAPDH*; 3, *ACTIN*; 4, *UBI*; 5, *TUB*; 6, *TIP41*; 7, *CACS*; 8, *PP2A*; 9, *TUA*; 10, *eIF4A*; 11, *CYP*; 12, *TCTP*; 13, *ABC*; 14, *F-BOX*; 15, *CAT*.

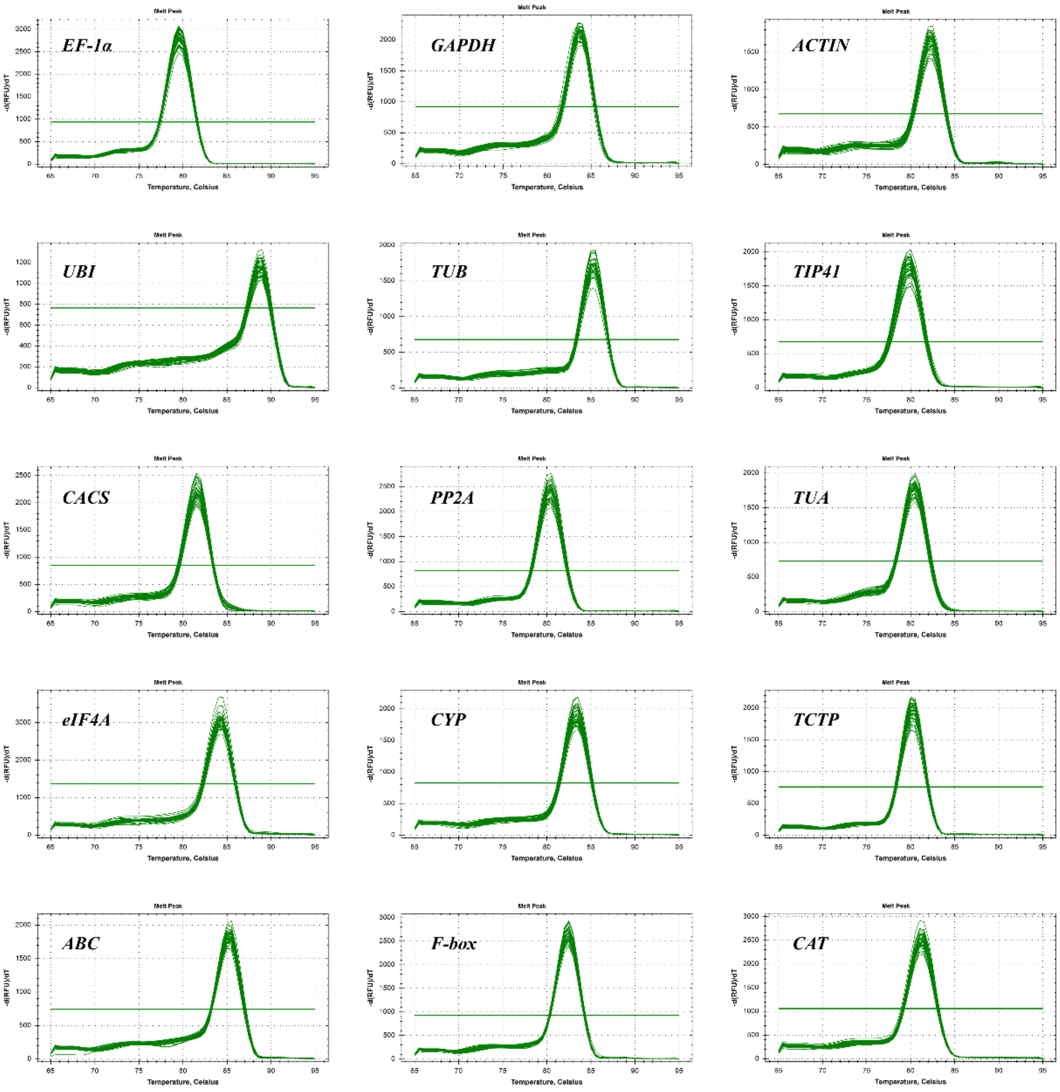

Figure S2. Melting curves of the fourteen candidate reference genes and target gene.

Table S1. Description of 14 candidate reference genes and one target gene.

Gene name	Gene symbol	Alias Gene Symbol	Homolog locus	E value	Total score	Identity (%)
Elongation factor 1-alpha	*EF1A*	LOC109758469	XM_020317327	0.0	2614	95.62
glyceraldehyde-3-phosphate dehydrogenase 2, cytosolic	*GAPDH*	LOC109753918	XM_020312837	0.0	2011	97.22
Actin-1	*ACT1*	LOC109753876	XM_020312803	0.0	2623	97.23
Polyubiquitin-like	*UBI*	LOC119324515	XM_037598302	0.0	1725	94.51
Tubulin beta-3 chain	*TUBB3*	LOC109735803	XM_020295003	0.0	2652	97.15
TIP41-like protein	*TIPRL*	LOC109743484	XM_020302576	0.0	1565	92.83
Clathrin adaptor complex subunit	*CACS*		KX268090	0.0	1796	90.85
serine/threonine-protein protein phosphatase 2A 65 kDa regulatory subunit A beta isoform	*PPP2R1B*	LOC119301141	XM_037578045	0.0	3640	97.52
Tubulin alpha-1 chain	*TUBA1A*	LOC109750814	XM_020309759	0.0	2772	96.21
Eukaryotic initiation factor 4A	*EIF4A1*	LOC109751838	XM_020310718	0.0	3086	98.35
Cyclophilin A-3	*CYPA3*		AF262984	0.0	1212	93.13
Translationally controlled tumor protein	*TCTP*		AF508970	0.0	1214	94.98
ABC transporter G family member 11-like	*ABCG11L*	LOC119355790	XM_037622666	0.0	4087	96.50
F-box only protein 6-like	*FBXO6L*	LOC119278203	XM_037559556	0.0	2946	95.57
Catalase-1	*CAT1*	LOC119291874	XM_037570687	0.0	2990	96.09
